# Live Simultaneous Monitoring of Mineral Deposition and Lipid Accumulation in Differentiating Stem Cells

**DOI:** 10.3390/biomimetics4030048

**Published:** 2019-07-10

**Authors:** Nigel De Melo, Sarah McGinlay, Robert Markus, Laura Macri-Pellizzeri, Michael E. Symonds, Ifty Ahmed, Virginie Sottile

**Affiliations:** 1 Wolfson STEM Centre, School of Medicine, The University of Nottingham, Nottingham NG7 2RD, UK; 2 School of Life Sciences, University of Nottingham, Nottingham NG7 2RD, UK; 3 The Early Life Research Unit, Division of Child Health, School of Medicine, The University of Nottingham, Nottingham NG7 2RD, UK; 4 Advanced Materials Group, Faculty of Engineering, The University of Nottingham, Nottingham NG7 2RD, UK; 5 Arthritis Research UK Pain Centre, The University of Nottingham, Nottingham NG7 2RD, UK

**Keywords:** live monitoring, stem cell, mineralisation, lipid detection

## Abstract

Mesenchymal stem cells (MSCs) are progenitors for bone-forming osteoblasts and lipid-storing adipocytes, two major lineages co-existing in bone marrow. When isolated in vitro, these stem cells recapitulate osteoblast or adipocyte formation if treated with specialised media, modelling how these lineages interact in vivo. Osteogenic differentiation is characterised by mineral deposits accumulating in the extracellular matrix, typically assessed using histological techniques. Adipogenesis occurs with accumulation of intracellular lipids that can be routinely visualised by Oil Red O staining. In both cases, staining requires cell fixation and is thus limited to end-point assessments. Here, a vital staining approach was developed to simultaneously detect mineral deposits and lipid droplets in differentiating cultures. Stem cells induced to differentiate produced mixed cultures containing adipocytes and bone-like nodules, and after two weeks live cultures were incubated with tetracycline hydrochloride and Bodipy to label mineral- and lipid-containing structures, respectively. Fluorescence microscopy showed the simultaneous visualisation of mineralised areas and lipid-filled adipocytes in live cultures. Combined with the nuclear stain Hoechst 33258, this approach further enabled live confocal imaging of adipogenic cells interspersed within the mineralised matrix. This multiplex labelling was repeated at subsequent time-points, demonstrating the potential of this new approach for the real-time high-precision imaging of live stem cells.

## 1. Introduction

Bone marrow contains a range of hematopoietic and mesenchymal cell types, including stem cell populations that can be isolated and differentiated in vitro. Among them, mesenchymal stem cells (MSCs) are multipotent progenitors able to generate bone, adipose and cartilage cell types. Under defined culture conditions, MSCs can be induced to form osteoblasts and chondrocytes, two cell types of biomedical importance for strategies aiming to address skeletal damage [1,2,3]. MSCs have also been used to generate adipocytes, providing an efficient in vitro model to characterize factors regulating adipose tissue differentiation [4,5].

MSC differentiation has become a topic of increasing importance in the context of bone physiology, as several studies have suggested a link between bone health and the adipocyte content of the marrow, referred to as fatty marrow [5,6,7,8,9,10]. Since MSCs can form both osteogenic and adipogenic lineages, they represent an important resource for studying the regulation of bone versus adipose cell formation, which can be modelled in vitro [11,12]. Established culture protocols can induce efficient MSC differentiation, which is routinely assessed using endpoint staining techniques. Osteogenic differentiation is characterised by the accumulation of mineral deposits in the extracellular matrix, which can be analysed by Alizarin Red S staining. Adipogenic differentiation is accompanied by the progressive formation of intracellular lipid droplets, which are easily detected by Oil Red O staining. However, these two techniques require cells to be fixed, and are thus not compatible with live measurements. Some additional dyes have become available to stain cells without the need to sacrifice cultures. In the field of adipogenesis, Bodipy 493/503 (4,4-difluoro-1,3,5,7,8-pentamethyl-4-bora-3a,4a-diaza-*s*-indacene) has been shown to enter live cells and fluorescently stain lipid inclusions [13]. In parallel, we recently developed a live fluorescent labelling method based on short incubation with the antibiotic molecule tetracycline for the visualization of mineral deposits in osteogenic cultures [14].

In an attempt to jointly monitor the adipogenic and osteogenic lineages appearing concomitantly in MSC cultures, we have developed a simple new approach using vital labels that enables the real-time monitoring of multiple lineages appearing during stem cell differentiation in culture.

## 2. Materials and Methods

All reagents were purchased from ThermoFisher Scientific (Loughborough, UK) unless otherwise stated.

### 2.1. Cell Culture and Differentiation 

Mouse MSCs (D1, ATCC CRL-12424) were seeded at a density of 75,000 cells/cm^2^ on 24-well culture plates in standard medium (SM) (low-glucose Dulbecco’s modified Eagle’s medium supplemented with 10% foetal calf serum, 1% non-essential amino acids, 1% L-glutamine and 1% penicillin/streptomycin). After 12 h, medium was changed to differentiation medium (DM) prepared by supplementing SM with 10 mM β-glycerophosphate, 50 μM ascorbic acid and 0.1 μM dexamethasone. Parallel wells kept in SM were used as undifferentiated controls. Cells were maintained at 37 °C and 5% CO_2_, with medium refreshed every 48 h until the endpoint (day 25).

### 2.2. Fluorescence Labelling and Imaging 

Cells were labelled and imaged live at various time-points as indicated. Matrix mineralization was stained with tetracycline hydrochloride (TC, 20 μg/mL) (Sigma-Aldrich, Gillingham, UK) as previously described [14]. Lipids were stained by 30-min incubation with Bodipy 493/503 (BD, 0.5 μg/mL). Cell nuclei were stained with Hoechst 33,258 (HT, 20 μg/mL) for 10 min. After staining, cells were washed in phosphate-buffered saline (PBS) and medium was refreshed. Fluorescence and bright field images were taken on a Nikon Eclipse TS2 microscope using C-LED 470 and C-LED 385 filter cubes coupled to a Nikon D3300 camera (Kingston upon Thames, UK). Signal quantification of TC and BD staining was quantified in-well at the endpoint on an Infinite 200 (Tecan, Reading, UK) plate reader (excitation/emission for TC: 405/557 nm, for BP: 455/510 nm) after fixation (data presented as mean ± SEM for duplicate wells with 25 reads/well).

### 2.3. Confocal Microscopy and 3D Imaging

Cells were imaged using a Zeiss 10×/0.45 NA water immersion objective on a Zeiss Elyra PS1 LSM780 inverted confocal microscope (Zeiss, Cambridge, UK). Spherical and chromatic aberration (image stretching in Z and channel mismatch) was assessed by scanning 4 μm TetraSpeck beads (TetraSpeck™ Fluorescent Microspheres, ThermoFisher cat. no: T7284). Optical aberration data was extracted (Channel Alignment protocol in Zeiss Zen 2012 software) and a correction value was applied to achieve a spherical view of the beads in the Z dimension. These corrections were then applied to the 3D rendered images. Channels were scanned independently (sequential scan) to avoid crosstalk. Confocal settings were as follows: excitation/emission for TC was 405/494–531 nm, for BD was 488/535–579 nm and for HT was 405/420–457 nm). As TC and BD absorbed different excitation lasers while emitting in the same range, red pseudo-colouring was applied to the TC signal for clarity.

### 2.4. Cell Metabolic Assay 

Metabolic activity was measured in triplicate at day 25 using a PrestoBlue Cell Viability kit according to the manufacturer’s instructions. Cells were incubated with the Presto Blue solution for 30 min at 37 °C and 5% CO_2_, and the fluorescence was then quantified on an Infinite 200 (Tecan) plate reader (560/590 nm excitation/emission). Data was presented as mean ± SEM (*n* = 3).

### 2.5. Alizarin Red and Oil Red O Staining 

Before lipid and matrix mineralisation quantification at the endpoint, cells were fixed with 4% paraformaldehyde (PFA) and washed with PBS. Matrix mineralisation was stained by adding 200 μL of 1% *w/v* Alizarin Red S solution to each well and incubating for 15 min at room temperature. Excess stain was washed with deionized water and plates were imaged on a Nikon Eclipse TS2 microscope. Oil Red O staining was performed as previously described [4].

Data was analysed using GraphPad Prism Software 7.04 (GraphPad Software Inc., San Diego, CA, USA), and a Student’s *t*-test was used for the comparison of two groups.

## 3. Results

**Figure 1:**
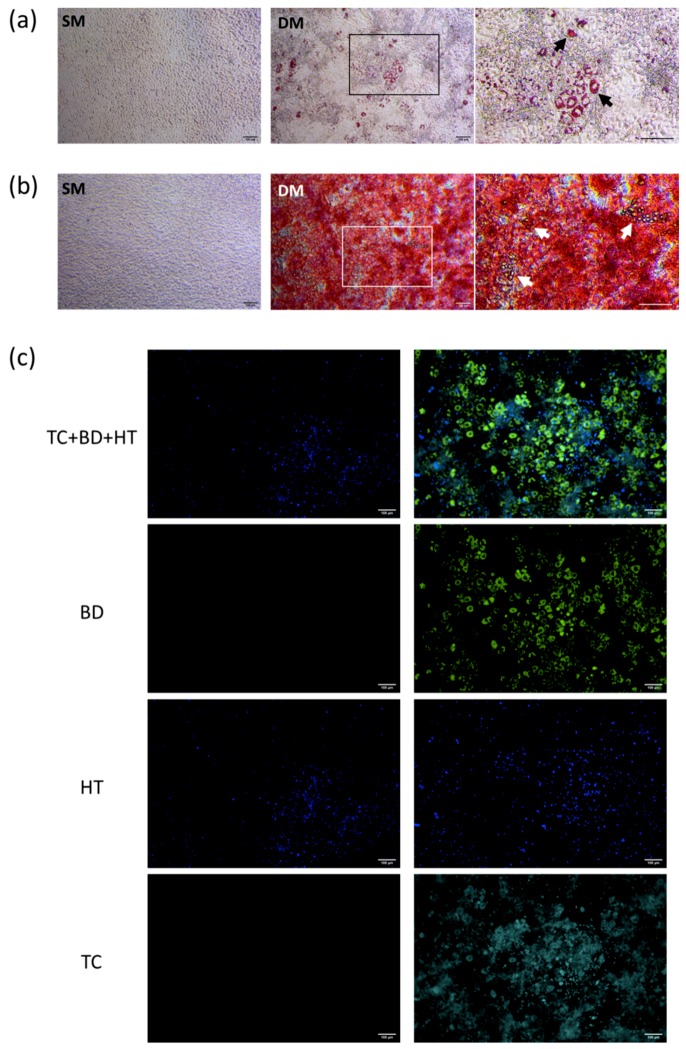
Imaging of MSC cultures under control and differentiating culture conditions. (**a**,**b**) Typical validation of dual MSC differentiation at the endpoint showing strong Oil Red O-positive lipids (**a**) and Alizarin Red-positive signal (**b**) under DM treatment, revealing adipocytes (arrows) embedded in the strongly mineralised matrix. Scale bar = 100 μm (**c**) Fluorescence imaging of live MSCs after combined TC, BD and HT labelling at day 19 under undifferentiated (left panel) and differentiated (right panel) conditions, showing strong BD intensity (bright green) with diffuse TC signal (turquoise) with HT nuclear counterstain (blue). Representative images taken from two cultures stained in parallel. Scale bar = 100 μm.

In order to establish a combined lineage monitoring approach for differentiation, MSCs were seeded in culture and treated with SM (standard medium), or DM (differentiation medium) able to promote both adipogenic and osteogenic lineages. DM treatment induced a strong differentiation response as demonstrated by the joint presence of lipid droplets and mineral deposits, validated by Oil Red O and Alizarin Red staining, respectively (Figure 1a,b).

To test the combined labelling approach, live cultures were incubated with TC, BD and HT before observation under a fluorescence microscope, which revealed strong differences between SM and DM conditions visible at day 19 (Figure 1c). While undifferentiated cells in SM were not positively stained by TC and BD as expected, the differentiated DM cultures showed intense BD signal in intracellular lipid droplets, surrounded by diffuse TC signal throughout the monolayer.

**Figure 2:**
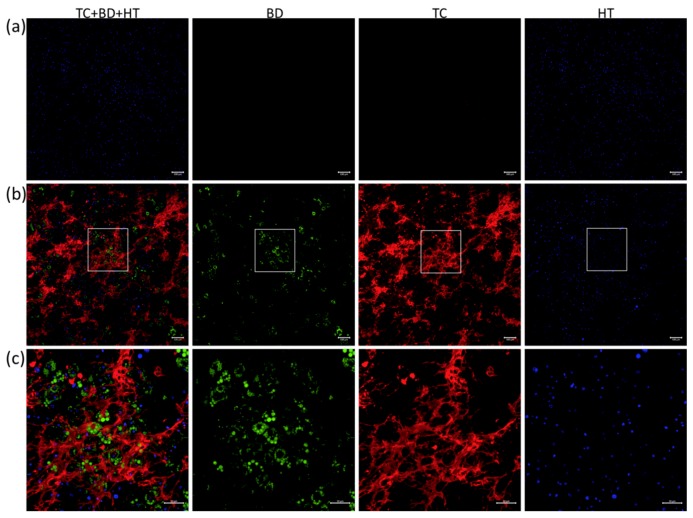
Confocal imaging of MSC cultures after combined TC, BD and HT labelling at day 21. Culture under undifferentiated (**a**) and differentiated (**b**,**c**) conditions observed using confocal microscopy showed specific signalfrom BD (green) and TC (red) in differentiated cultures, which could be combined with HT (blue) nuclear staining. Scale bar = 100 μm. (**c**) High-magnification view of the differentiated cells. Scale bar = 50 μm.

Confocal microscopy in sequential scanning mode was then exploited to improve detection by separating the labels to better differentiate lipid and mineralisation signals. This produced a clear TC signal that highlighted hotspots of mineral deposition in the extracellular matrix. Cells with strong BD signal, indicative of intracellular lipids, were present throughout the matrix in DM-treated cultures (Figure 2). In contrast, the combined labelling of undifferentiated cells only produced HT signal confirming the specificity of the vital staining approach. No BD signal was visible in the TC channel.

**Figure 3:**
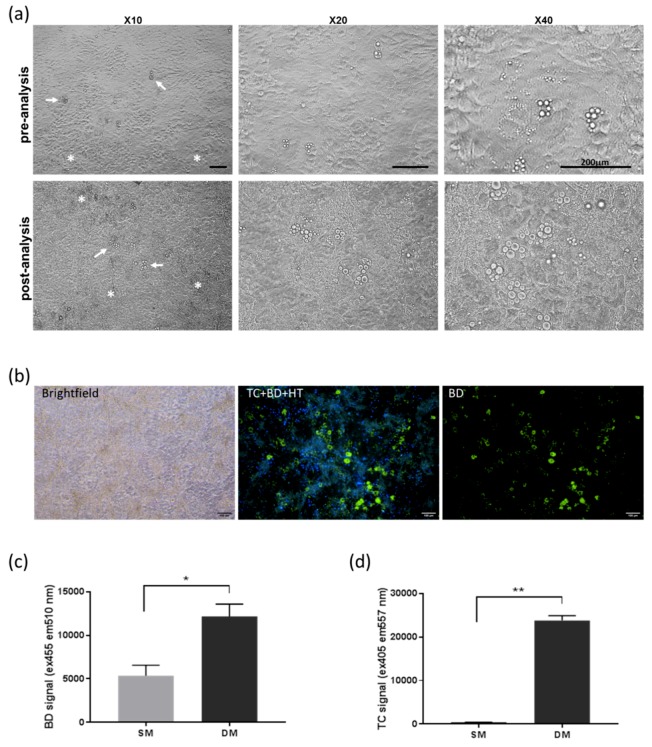
MSC cultures observed before and after TC/BD/HT labelling. (**a**) Brightfield images of differentiated MSCs before (day 10) and after (day 23) undergoing live labelling and imaging steps, confirming maintained differentiation phenotypes as indicated by mineral deposits (asterisks) and adipocytes (arrows). Scale bar = 200 μm (**b**) Fluorescence imaging of live cultures repeated at the endpoint, confirming sustained differentiation 6 days after the initial labelling and imaging carried out at day 19. Scale bar = 100 μm. (**c**,**d**) Corresponding signal quantification using a plate-reader showing increased BD and TC signal in DM conditions (* *p* < 0.05, ** *p* < 0.005).

Cultures observed on days following the live labelling and imaging steps maintained their differentiated phenotype (Figure 3a). To establish whether this multiplexed labelling procedure could enable repeated imaging over time to monitor differentiation, stained cells were re-imaged at the day 25 endpoint (Figure 3b), which confirmed that this procedure could be used to re-assess cells over the differentiation period. TC and BD fluorescence was also analysed directly in the culture plates at the endpoint using a plate reader, displaying a clear difference in TC intensity between SM and DM conditions (Figure 3c,d), while the metabolic activity was unchanged (Figure S1).

**Figure 4:**
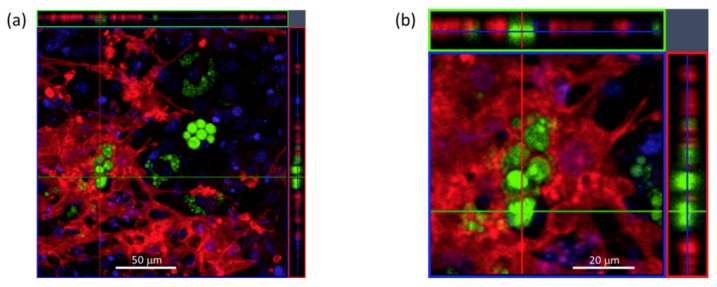
Confocal 3D imaging of differentiated MSC cultures after combined TC/BD/HT labelling at the endpoint. (**a**) Orthogonal views showing BD-positive adipocytes containing intracellular lipid droplets (green) surrounded by the TC-positive mineralised matrix (red), with HT (blue) nuclear counterstain, shown at higher magnification (**b**). Scale bar = 50 μm (**a**) and 20 μm (**b**). See additional 3D reconstruction in Supplementary Materials (Video S1 and S2).

Confocal *z*-stack images were taken at the endpoint to better observe the close association of the mixed phenotypes obtained in differentiated MSC cultures (Figure 4). These revealed lipid-filled adipocytes, marked by strong cytoplasmic BD signal, interspersed and embedded in the TC-labelled mineralised matrix.

## 4. Discussion

Multipotent stem cells can recapitulate in vitro the formation of osteogenic and adipogenic lineages found in bone marrow [10]. Here, a combined labelling approach was developed to enable the monitoring of these two lineages simultaneously in live cultures through fluorescence imaging. The present approach successfully combined three vital stains applied to differentiating cultures to label lipid droplets, the mineralised matrix and cell nuclei at once, to achieve high-resolution imaging in live cells.

The progressive mineral deposition observed in cultures undergoing osteogenesis has historically been evaluated using endpoint assays performed on fixed cells, such as Alizarin Red or Von Kossa staining, detecting calcium phosphate ions [15,16,17]. Similarly, the appearance of lipid-filled droplets in cells undergoing adipogenesis has traditionally been documented using lipophilic dyes such as Nile Red or Oil Red O as endpoint assays [15,17,18]. Immunodetection of specific lineage markers is also widely used as an endpoint phenotyping procedure, and advanced analytical techniques such as Raman spectroscopy have also recently been applied to stem cell cultures as a way to detect mineral-rich or lipid-rich areas in vitro [19,20,21].

Beyond common staining procedures relying on fixed cells, vital labelling has been introduced for the separate monitoring of adipogenic and osteogenic lineages, but not yet established for both lineages simultaneously. We recently reported that TC could be used for the live labelling and quantitative analysis of MSC cultures undergoing osteogenic differentiation [14], and BD has been used to label neutral lipids in both fixed and live cells for imaging- and flow cytometry-based analyses [22,23,24]. Both stains have been shown not to impair the appearance of the target lineages [13,14].

Notably, the approach developed here provides a non-disruptive alternative to the use of flow cytometry, which, although very useful for acquiring large quantitative datasets, requires the disruption of cell monolayers in order to recover single cells [25,26]. This poses substantial problems if analysing both adipogenic and osteogenic lineages, since the cell recovery protocols required for flow cytometry can cause damage to mature adipocytes and destruction of the extracellular matrix. By contrast, the present live-imaging approach alleviates the need to disrupt cells as they differentiate, providing a simple solution for analysing differentiated cells in situ.

This ability to label cells live without disruption further demonstrates the validity of this approach for the longitudinal monitoring of cultures in real-time by fluorescence imaging. The same culture can be re-analysed over consecutive time-points, with possible imaging of the same field of view, to generate high-quality images that reflect the differentiation kinetics over the treatment period.

The compatibility with live confocal imaging is particularly useful, since it enables spectral imaging to separate lasers and emissions and minimise signal overspill. Application of confocal microscopy to this multiplex labelling approach can thus uniquely achieve live monitoring of cell–cell and cell–matrix interactions known to regulate mesenchymal differentiation [27,28,29]. Automated image analysis applied to these high-resolution staining images could be further developed to extract quantitative information on the levels of lipid and mineral signal distributed throughout the cultures.

Further developments of this technique could be envisaged through the use of additional live fluorescent labels compatible with those established here. Multiple label quantitation, which is readily accessible by measuring signal intensity through advanced image analysis tools, could also be further developed using spectrophotometry, as possible for each individual label [14,30]. Observations made here suggest compatibility between lipid- and mineral-specific labels, and therefore support the possible extension of this multiplex approach to in-well quantification. The applicability of this live-labelling approach thus offers a new non-disruptive modality to perform high-precision monitoring of multilineage differentiation concomitantly, in real time, which will be of particular benefit for biomedical and stem cell differentiation studies.

## Supplementary Materials

The following are available online at https://www.mdpi.com/2313-7673/4/3/48/s1, Figure S1: Metabolic activity assay carried out at day 25. VideoS1: 3D reconstruction of confocal analysis of DM-treated cells after TC, BD, HT labelling at endpoint. VideoS2: 3D reconstruction of confocal analysis under similar conditions, imaged at a higher magnification.
